# Adverse events of hepatic anti-fibrotic agents in phase 3 and above clinical trials: a descriptive analysis of the WHO-VigiAccess database

**DOI:** 10.3389/fphar.2025.1534628

**Published:** 2025-01-24

**Authors:** Yuwei Liu, Xu Zhao, Xinrui Wang, Qiang Zhou

**Affiliations:** ^1^ Department of Hepatology, Center of Infectious Diseases and Pathogen Biology, The First Hospital of Jilin University, Changchun, Jilin, China; ^2^ Key Laboratory of Zoonosis Research, Ministry of Education, The First Hospital of Jilin University, Changchun, China

**Keywords:** liver fibrosis, adverse drug reactions (ADRs), anti-fibrotic agents, WHO-VigiAccess, descriptive analysis, disproportionality analysis

## Abstract

**Introduction:**

Liver fibrosis is a pathological condition in response to chronic liver injuries. Currently, there is no Food and Drug Administration (FDA) approved pharmacotherapy for liver fibrosis. Advances in understanding hepatic fibrogenesis have led to the development of anti-fibrotic agents, and some of them have shown promise in phase 3 and above clinical trials. However, adverse drug reactions (ADRs) associated with emerging anti-fibrotic agents may hinder their efficacy and clinical applicability. This study assessed ADRs associated with anti-fibrotic agents as reported in the World Health Organization (WHO) VigiAccess database and compared the adverse reaction characteristics of these agents for optimizing therapeutic strategies.

**Methods:**

A detailed search was conducted on ClinicalTrial.gov to identify phase 3 or 4 clinical trials involving hepatic anti-fibrotic agents. The ADR reports were retrieved from the WHO-VigiAccess database, with data categorized by demographic characteristics, geographic distribution, and System Organ Classes (SOCs). The most frequently reported ADRs were identified through descriptive analysis. Disproportionality analysis, measured by reporting odd ratio (ROR) and proportional reporting ratio (PRR), was performed to evaluate ADRs related to gastrointestinal disorders.

**Results:**

Five hepatic anti-fibrotic agents (empagliflozin, liraglutide, candesartan, obeticholic acid, and resmetirom) were identified. A total of 130,567 ADR reports were analyzed, with empagliflozin, liraglutide, and candesartan showing significantly higher ADRs. The most frequently reported SOCs included gastrointestinal disorders (29.44%), general disorders (24.12%), and nervous system disorders (14.42%). Liraglutide demonstrated a higher risk of gastrointestinal ADRs (ROR: 4.629, 95% CI: 4.517–4.744; PRR: 3.566, 95% CI: 3.492–3.642) compared to the other agents. Severe ADRs were reported in empagliflozin, such as ketoacidosis and infections, while liraglutide was associated with pancreatitis and candesartan with acute kidney injury. Serious ADR rates varied, with candesartan reporting the highest proportion (7.28%).

**Conclusion:**

While hepatic anti-fibrotic agents showed promise in addressing liver fibrosis, their ADR profiles underscore the importance of pharmacovigilance and personalized treatment approaches. Future efforts should focus on improving the pharmacovigilance system, expanding population diversity in trials, and conducting ongoing research and extensive post-marketing surveillance.

## Introduction

Liver fibrosis is a pathological condition with the formation of fibrous scar in response to persistent liver injury caused by viral infection, alcohol abuse, non-alcoholic fatty liver disease (NAFLD), non-alcoholic steatohepatitis (NASH), and biliary obstruction. Regardless of the etiologies, persistent liver fibrosis can lead to distorted hepatic architecture, impaired liver function, cirrhosis, and ultimately, liver failure or hepatocellular carcinoma (HCC), which imposes significant clinical and economic burdens worldwide. Despite its reversible nature during early stages, effective pharmacological interventions targeting hepatic fibrosis remain an unmet medical need (Kisseleva and Brenner, 2021).

Currently, there is no Food and Drug Administration (FDA) approved pharmacotherapy for liver fibrosis. However, advancements in our understanding of hepatic fibrogenesis have spurred the development of anti-fibrotic agents aimed at modulating pathways involved in inflammation, hepatic stellate cell (HSC) activation, oxidative stress, and extracellular matrix remodeling (Tsuchida and Friedman, 2017; Parola and Pinzani, 2019; Hammerich and Tacke, 2023). Targeting these pathways, the small molecular inhibitors or agonists, are promising candidates for alleviating fibrosis, slowing disease progression, and improving clinical outcomes. In March 2024, the thyroid hormone receptor beta (THR-β) selective agonist resmetirom gained conditional approval from FDA as the first pharmacologic treatment for NASH patients with moderate-to-advanced liver fibrosis (FDA, 2024). In a phase 3 trial, resmetirom was superior to placebo in NASH resolution and improvement in hepatic fibrosis (Harrison et al., 2024). Glucagon-like peptide 1 (GLP-1) receptor agonists have been widely used to treat obesity and type 2 diabetes mellitus (T2DM). It has been shown that GLP-1 receptor treatment decreased histological inflammation and fibrosis in NASH patients (Nahra et al., 2021; Tan et al., 2022; Sanyal et al., 2024).

The major concern of hepatic anti-fibrotic agents is their adverse effects that may limit their efficacy and broad applicability. A comprehensive evaluation of these side effects is critical to balancing therapeutic benefits against potential risks and ensuring safe, patient-centered care. Therefore, pre-clinical tests and clinical trials are promising and urgently required. Assessing drug safety based on real-world large sample data is also worth further study. Currently, using spontaneous reporting systems (SRS) to collect real-world medication safety data is considered a more credible and reliable approach. The Uppsala Monitoring Center (UMC), representing the World Health Organization (WHO) programme for International Drug Monitoring (PIDM), has gathered global data on adverse drug reactions (ADRs) and developed the VigiBase database. Altogether, the WHO programme members today represent nearly 99% of the world’s population and contribute data to VigiBase with around 40 million ADRs (http://who-umc.org/vigibase/).

The hepatic anti-fibrotic agents in ongoing clinical research, especially in phase 3 and above clinical trials, showed good efficacy characteristics and are the most promising medications to reach the market for the treatment of liver fibrosis. In the present study, we retrieved hepatic anti-fibrotic agents in phase 3 and above clinical trials registered in ClinicalTrials.gov and performed a descriptive analysis of ADR reports in the WHO-VigiAccess database. We also conducted the disproportionality analysis to assess the gastrointestinal ADRs of these agents, as gastrointestinal disorders are the most common side effects of anti-fibrotic agents. By analyzing these data, we seek to highlight current challenges in antifibrotic drug development and outline future directions for achieving safer and more effective therapies for liver fibrosis.

## Materials and methods

### Identify target hepatic anti-fibrotic agents

ClinicalTrial.gov is an authoritative database of clinical research, which is maintained by the National Library of Medicine. The website (http://clinicaltrials.gov) was searched in detail to find clinical trials of hepatic anti-fibrotic agents registered before October 30, 2024. The research focus of “Condition/disease” comprised the terms “liver fibrosis” OR “hepatic fibrosis”. Manual searches were performed based on electronic searches as a supplement. Clinical trials were included if they met the following criteria: (1) study phase: phase 3 or phase 4; (2) study status: recruiting or completed with results; (3) study type: interventional studies. Then, the whole protocols were screened manually of which studies met the inclusion criteria. Finally, five anti-fibrotic agents “Empagliflozin”, “Liraglutide”, “Candesartan”, “Obeticholic acid”, “Resmetirom” were identified.

### Data sources

The WHO-VigiAccess database (https://www.vigiaccess.org) was searched on November 1, 2024, for all reported ADRs following the above five anti-fibrotic agent treatments. Data were collected among age, groups, sex, report year, and continents of the world by WHO-VigiAccess.

WHO-VigiAccess is a publicly accessible online platform that provides access to data from Vigibase. It is designed to promote transparency and facilitate research by offering insights into potential ADRs reported by healthcare professionals, patients, and regulatory authorities from over 150 countries. The definition relied on system organ classes (SOCs) and preferred terms (PTs) by the Medical Dictionary for Regulatory Activities (MedDRA) and WHODrug. Thus, records on each anti-fibrotic agent were retrieved, and all individual ADRs based on MedDRA SOC and PT levels recorded were identified to describe the spectrum of toxicities. A total of 27 items were classified by SOC and analyzed in the present study. Moreover, we focused on the PTs to identify the most common ADRs of each anti-fibrotic agent. According to outcome codes, three severity categories were defined to evaluate the safety results: death, hospitalization, and major events, including life-threatening events, disabilities, and congenital abnormalities.

### Disproportionality analysis

In pharmacovigilance study, disproportionality emerges when a specific ADR is associated with a given drug. The aim of disproportionality analysis is to identify statistical associations between a specific drug and ADRs of interest (Bate and Evans, 2009). The reporting odds ratio (ROR) (van Puijenbroek et al., 2002) and the proportional reporting ratio (PRR) (Evans et al., 2001) are the two most commonly used metrics for disproportionality analysis. They are calculated to measure the likelihood of imbalance of reporting an ADR for a specific drug in comparison to other drugs. The calculation formula of ROR and 95% confidence interval (CI) is as follows:

$$\begin{array}{c} \text{ROR}=\frac{a\times d}{c\times d}\\ 95\%\text{ CI}=e^{\sqrt[{In\left(ROR\right)\pm 1.96}]{\frac{1}{a}+\frac{1}{b}+\frac{1}{c}+\frac{1}{d}}} \end{array}$$

Where a, b, c, and d indicated the number of reports for the specific drug with specific ADR, the specific drug with other ADRs, the other drugs with specific ADR, and the other drugs with other ADRs, respectively. If the ROR value is greater than 2 (ROR >2) and the lower limit of the 95% CI is greater than one at the same time, with at least three cases, the ROR is considered significant and there may be disproportionate and potentially indicative of a safety risk. PRR is another index that measures the disproportionality of ADR reports. It is calculated as:

$$\begin{array}{c} \text{PRR}=\frac{a/\left(a+b\right)}{c/\left(c+d\right)}\\ 95\%\text{ CI}=e^{\sqrt[{\mathrm{In}\;\left(PRR\right)\pm 1.96}]{\frac{1}{a}+\frac{1}{a+b}+\frac{1}{c}+\frac{1}{c+d}}} \end{array}$$

When the value of the PRR is greater than 2 with at least three cases, the PRR was considered significant. These criteria ensure that the observed disproportionality is not the result of random variability. By using ROR and PRR in our research, we were able to thoroughly evaluate the disproportionality of gastrointestinal issues linked to hepatic anti-fibrotic agent use.

### Statistical analysis

The descriptive analysis was performed to illustrate the characteristics of ADRs associated with the five hepatic anti-fibrotic agents. Descriptive variables were categorized using frequency and percentage. All analyses were conducted using R programming software (version 4.4.0), with statistical significance set at a *p*-value of less than 0.05. Forest plots were created using the “forestplot” R package (Version 3.1.5).

## Results

### Basic information on five hepatic anti-fibrotic agents

**FIGURE 1:**
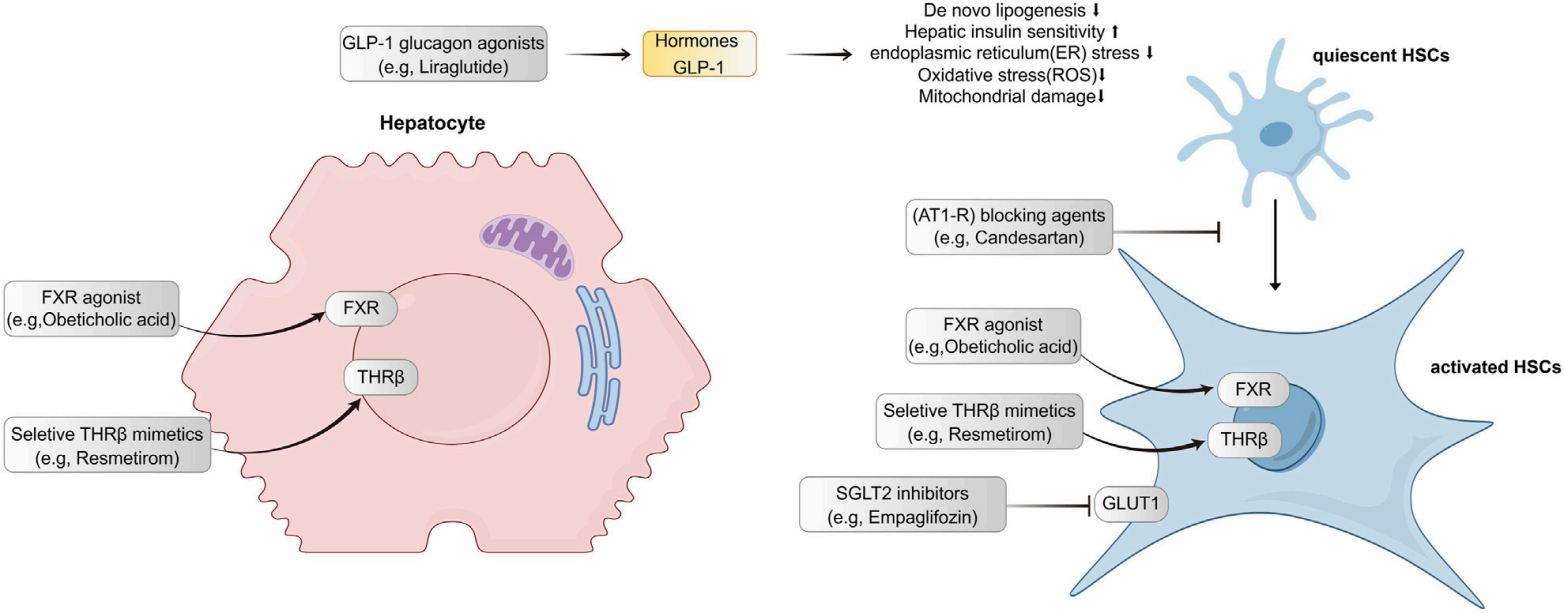
Targets and mechanisms of action for five hepatic-antifibrotic agents. Their regulation pathways are mainly associated with hepatocyte injuries and hepatic stellate cell activation. GLP-1, Glucagon-like peptide 1; FXR, farnesoid X receptor; THR, thyroid hormone receptor; GCGR, glucagon receptor; HSC, hepatic stellate cell; AT1-R, angiotensin II type 1 receptor; SGLT2, sodium-glucose co-transporter 2; GLUT1, glucose transporter type 1.

Table 1 shows the basic information of five hepatic anti-fibrotic agents that we have studied for clinical research. Figure 1 illustrates their unique mechanisms of action. Each of the agents has a unique molecular target. Empagliflozin is a kind of sodium-glucose co-transporter 2 (SGLT2) inhibitor, which could attenuate HSC activation and fibrogenesis (Shen et al., 2023). Studies have demonstrated that empagliflozin improves liver fibrosis in patients with NAFLD without T2DM (Taheri et al., 2020; Takahashi et al., 2024). It is now in the phase 4 clinical trial for the treatment of liver fibrosis. Liraglutide is a kind of long-acting GLP-1 analogue. It has been shown to reduce insulin resistance, oxidative stress, and mitochondrial damage as well as improve liver histology (Armstrong et al., 2016). Its effects were evaluated during phase 4 period for NAFLD patients with liver fibrosis. The renin-angiotensin system can be an attractive hepatic anti-fibrotic target. The effects of candesartan, a kind of angiotensin II type 1 receptor (AT1-R) blocking (ARB) agent have been explored in patients with alcoholic liver fibrosis (Kim et al., 2012). Currently, a phase 3 clinical trial is ongoing to evaluate its efficacy on liver fibrosis in patients with chronic hepatitis C. Obeticholic acid, a farnesoid X receptor (FXR) agonist, has been shown to improve the liver histological features of NASH patients (Younossi et al., 2019). The phase 3 study evaluating the efficacy of obeticholic acid in cirrhotic patients due to NASH has been completed. Resmetirom is an oral, liver-directed, selective THR-β agonist that showed efficacy in NASH resolution and improvement in liver fibrosis. The phase 3 study is ongoing to evaluate the effectiveness of resmetirom in NASH patients with compensated cirrhosis, and the latest results showed that it was effective in these patients. Patients with F2–F3 fibrosis will be enrolled in the advanced research.

**TABLE 1:** General information for five hepatic anti-fibrotic agents.

Drug name	Structure	Chemical formula	Target	Phase of clinical trial	Clinicaltrials.gov number	Ref
Empagliflozin	SGLT2 inhibitors	C23H27ClO7	SGLT2	Phase 4	NCT05147090	Taheri et al. (2020)
Liraglutide	GLP-1 analogues	C172H265N43O51	GLP-1	Phase 4	NCT06501326	Armstrong et al. (2016)
Candesartan	AT1-R blockers (ARB)	C24H20N6O3	AT1-R	Phase 3	NCT03770936	Kim et al. (2012)
Obeticholic acid	FXR agonists	C26H44O4	FXR	Phase 3	NCT03439254	Younossi et al. (2019)
Resmetirom	THR beta agonists	C17H12Cl2N6O4	THRβ	Phase 3	NCT05500222	Harrison et al. (2024)

Abbreviations: Ref, references; SGLT2, sodium-glucose co-transporter 2; GLP-1, Glucagon-like peptide 1; AT1-R, angiotensin II type 1 receptor; FXR, farnesoid X receptor; THR, thyroid hormone receptor.

### Overall characteristics of ADR reports for five anti-fibrotic agents

The earliest reports of empagliflozin, liraglutide, candesartan, obeticholic acid, and resmetirom were received in the WHO-VigiAccess database in 2014, 2000, 1998, 2017, and 2024, respectively. Up to now (by November 2024), the WHO has received 43,095, 59,268, 22,033, 6,047, and 124 ADR reports for these five agents, respectively, with a total of 130,567 reports. The numbers of ADR reports were 42,347 cases of empagliflozin, 58,984 cases of liraglutide, 21,896 cases of candesartan, 5,607 cases of obeticholic acid, and 124 cases of resmetirom. Among the 130,567 ADR reports related to the five anti-fibrotic agents shown in Table 2, except for 9,983 cases in which the sex was unknown, the number of women (74,554) who had ADRs was significantly greater than that of men (46,030), and the female-male ratio was 1.62:1 with a significant discrepancy. Excluding the unknown age reports, most of the age groups with the highest reported rates are between 45 and 64 years. Most of the ADR reports were from the Americas (53.58%), followed by Europe (31.05%). Table 2 also lists the reporting years for each of the studied medications.

**TABLE 2:** Characteristics of ADR reports for five anti-fibrotic agents.

	Empagliflozin	Liraglutide	Candesartan	Obeticholic acid	Resmetirom
Number of ADRs	43,095	59,268	22,033	6,047	124
Female	18,339 (42.55%)	40,588 (68.48%)	12,512 (56.79%)	3,114 (51.50%)	1 (0.81%)
Male	20,798 (48.26%)	16,170 (27.28%)	8,707 (39.52%)	352 (5.82%)	3 (2.42%)
Gender Unknown	3,958 (9.18%)	2,510 (4.24%)	814 (3.69%)	2,581 (42.68%)	120 (96.77%)
<18 years	89 (0.21%)	394 (0.66%)	166 (0.75%)	5 (0.08%)	—
18–44 years	1,880 (4.36%)	9,404 (15.87%)	1,400 (6.35%)	171 (2.83%)	1 (0.81%)
45–64 years	10,652 (24.72%)	19,396 (32.73%)	5,797 (26.31%)	1,051 (17.38%)	1 (0.81%)
65–74 years	8,223 (19.08%)	7,180 (12.11%)	4,574 (20.76%)	579 (9.57%)	2 (1.61%)
>75 years	5,450 (12.65%)	1,958 (3.30%)	4,745 (21.54%)	246 (4.07%)	—
Age unknown	16,801 (38.99%)	20,936 (35.32%)	5,351 (24.29%)	3,995 (66.07%)	120 (96.77%)
Before 2019	8,109 (18.82%)	28,377 (47.88%)	13,679 (62.08%)	2,433 (40.23%)	—
2019	5,515 (12.80%)	4,130 (6.97%)	2,105 (9.55%)	1,407 (23.27%)	—
2020	3,743 (8.69%)	3,363 (5.67%)	1,832 (8.31%)	656 (10.85%)	—
2021	4,294 (9.96%)	4,046 (6.83%)	1,144 (5.19%)	607 (10.04%)	—
2022	5,780 (13.41%)	5,760 (9.72%)	802 (3.64%)	335 (5.54%)	—
2023	7,697 (17.86%)	8,184 (13.81%)	1,234 (5.60%)	291 (4.81%)	—
Africa	155 (0.36%)	829 (1.40%)	173 (0.79%)	10 (0.17%)	—
Americas	21,904 (50.83%)	38,248 (64.53%)	4,570 (20.74%)	5,118 (84.64%)	124 (100%)
Asia	4,703 (10.91%)	7,311 (12.34%)	4,426 (20.09%)	8 (0.13%)	—
Europe	15,273 (35.44%)	12,272 (20.71%)	12,088 (54.86%)	906 (14.98%)	—
Oceania	1,060 (2.46%)	608 (1.03%)	776 (3.52%)	5 (0.08%)	—

Abbreviations: ADR, adverse drug reaction; “—” stand for not reported.

### Distribution of SOCs for five anti-fibrotic agents

Table 3 presents the reporting rates of 27 SOCs for five anti-fibrotic agents. Empagliflozin, liraglutide, and candesartan, due to their longer usage duration, have significantly higher incidence rates of diseases in various systems and organs than the other two novel anti-fibrotic agents. The five most common types of ADRs are as follows: gastrointestinal disorders (38,443 cases, 29.44%), general disorders and administration site conditions (31,487 cases, 24.12%), investigations indicating altered biochemical parameters (7,718 cases, 15.93%), nervous system disorders (18,831cases, 14.42%). The highest ADR report rates were reported as gastrointestinal disorders in liraglutide and resmetirom, whereas candesartan showed the highest ADR report rate related to general disorders and administration site conditions. The most common ADRs for empagliflozin were associated with Infections and infestations, and for obeticholic acid with skin and subcutaneous tissue disorders. Among the ADRs, there were seven types of SOCs for empagliflozin whose incidence rates exceeded 10%, five types for liraglutide, four types for candesartan, four types for obeticholic acid, as well as three types for resmetirom.

**TABLE 3:** ADR report numbers and rates of 27 SOCs for five anti-fibrotic agents.

System organ class	Empagliflozin (*N* = 43,095)	Liraglutide (*N* = 59,268)	Candesartan (*N* = 22,033)	Obeticholic acid (*N* = 6,047)	Resmetirom (*N* = 124)
Blood and lymphatic system disorders	264 (0.61%)	275 (0.46%)	408 (1.85%)	108 (1.78%)	1 (0.81%)
Cardiac disorders	1,994 (4.63%)	1,369 (2.31%)	1,488 (6.75%)	167 (2.76%)	1 (0.81%)
Congenital, familial and genetic disorders	203 (0.47%)	58 (0.10%)	150 (0.68%)	8 (0.13%)	0 (0%)
Ear and labyrinth disorders	423 (0.98%)	320 (0.54%)	550 (2.50%)	52 (0.86%)	1 (0.81%)
Endocrine disorders	72 (0.17%)	369 (0.62%)	94 (0.43%)	25 (0.41%)	0 (0%)
Eye disorders	823 (1.91%)	1,056 (1.78%)	783 (3.55%)	116 (1.92%)	1 (0.81%)
Gastrointestinal disorders	5,291 (12.28%)	28,252 (47.67%)	3,524 (15.99%)	1,315 (21.75%)	61 (49.19%)
General disorders and administration site conditions	6,655 (15.44%)	17,359 (29.29%)	5,449 (24.73%)	1,987 (32.86%)	37 (29.84%)
Hepatobiliary disorders	296 (0.69%)	1,189 (2.01%)	401 (1.82%)	519 (8.58%)	9 (7.26%)
Immune system disorders	285 (0.66%)	670 (1.13%)	385 (1.75%)	113 (1.87%)	3 (2.42%)
Infections and infestations	9,846 (22.85%)	2,155 (3.64%)	893 (4.05%)	433 (7.16%)	9 (7.26%)
Injury, poisoning and procedural complications	3,024 (7.02%)	0 (0%)	1,976 (8.97%)	846 (13.99%)	16 (12.90%)
Investigations	7,474 (17.34%)	9,816 (16.56%)	2,506 (11.37%)	948 (15.68%)	50 (40.32%)
Metabolism and nutrition disorders	8,335 (19.34%)	7,104 (11.99%)	1,762 (8.00%)	239 (3.95%)	5 (4.03%)
Musculoskeletal and connective tissue disorders	1,652 (3.83%)	2,251 (3.80%)	2,372 (10.77%)	564 (9.33%)	5 (4.03%)
Neoplasms benign, malignant and unspecified (incl cysts and polyps)	640 (1.49%)	2,184 (3.68%)	245 (1.11%)	125 (2.07%)	1 (0.81%)
Nervous system disorders	4,625 (10.73%)	8,282 (13.97%)	5,110 (23.19%)	789 (13.05%)	25 (20.16%)
Pregnancy, puerperium and perinatal conditions	9 (0.02%)	135 (0.23%)	157 (0.71%)	3 (0.05%)	0 (0%)
Product issues	109 (0.25%)	1,277 (2.15%)	838 (3.80%)	20 (0.33%)	0 (0%)
Psychiatric disorders	1,213 (2.81%)	2,736 (4.62%)	1,404 (6.37%)	413 (6.83%)	5 (4.03%)
Renal and urinary disorders	4,904 (11.38%)	1,443 (2.43%)	1,735 (7.87%)	204 (3.37%)	4 (3.23%)
Reproductive system and breast disorders	2,997 (6.95%)	702 (1.18%)	330 (1.50%)	33 (0.55%)	1 (0.81%)
Respiratory, thoracic and mediastinal disorders	1,525 (3.54%)	1,630 (2.75%)	2,724 (12.36%)	312 (5.16%)	2 (1.61%)
Skin and subcutaneous tissue disorders	4,116 (9.55%)	3,921 (6.62%)	3,424 (15.54%)	2,743 (45.36%)	22 (17.74%)
Social circumstances	165 (0.38%)	147 (0.25%)	101 (0.46%)	101 (1.67%)	2 (1.61%)
Surgical and medical procedures	1,106 (2.57%)	860 (1.45%)	142 (0.64%)	790 (13.06%)	0 (0%)
Vascular disorders	1,253 (2.91%)	991 (1.67%)	2,434 (11.05%)	198 (3.27%)	1 (0.81%)

Abbreviations: ADR, adverse drug reaction; SOC, system organ class.

### The most common ADR reports for five anti-fibrotic agents

The top 20 most reported ADRs of the five anti-fibrotic agents are presented in Table 4, and the manifestations listed were PTs from within the SOCs. The common ADRs of all five agents were nausea, diarrhoea, fatigue, and dizziness, and most of the ADRs were related to gastrointestinal disorders. The common ADRs for empagliflozin included some life-threatening events, such as ketoacidosis, infections (urinary tract infection and fungal infection), dehydration, and even death, which required extra vigilance. Most of the ADRs in the top 20 for the other four agents were minor events that are self-limiting. However, there are some noteworthy events, such as a higher reported rate of pancreatitis when using liraglutide, and a higher reported rate of acute kidney injury (AKI) when using candesartan.

**TABLE 4:** Top 20 ADRs of five anti-fibrotic agents.

Empagliflozin (*N* = 43,095)		Liraglutide (*N* = 59,268)		Candesartan (*N* = 22,033)		Obeticholic acid (*N* = 6,047)		Resmetirom (*N* = 124)	
ADR	Report rate (%)	ADR	Report rate (%)	ADR	Report rate (%)	ADR	Report rate (%)	ADR	Report rate (%)
Diabetic ketoacidosis	6.67	Nausea	20.84	Dizziness	4.70	Pruritus	40.04	Nausea	20.97
Urinary tract infection	5.26	Vomiting	9.96	Headache	3.30	Fatigue	12.01	Alanine aminotransferase increased	16.13
Fungal infection	4.89	Diarrhoea	8.85	Cough	2.46	Product dose omission issue	5.84	Aspartate aminotransferase increased	16.13
Weight decreased	4.52	Blood glucose increased	5.95	Nausea	2.20	Nausea	4.66	Diarrhoea	12.90
Blood glucose increased	3.89	Headache	5.67	Pruritus	2.12	Drug ineffective	4.18	Fatigue	12.90
Dizziness	3.61	Constipation	5.18	Hypotension	1.82	Arthralgia	4.18	Dizziness	12.90
Pollakiuria	3.18	Pancreatitis	4.45	Fatigue	1.82	Rash	4.02	Pruritus	12.90
Nausea	2.93	Decreased appetite	4.20	Dyspnoea	1.78	Constipation	3.64	Hepatic enzyme increased	11.29
Euglycaemic diabetic ketoacidosis	2.88	Dizziness	4.12	Myalgia	1.58	Dizziness	3.54	Abdominal pain upper	8.87
Rash	2.79	Abdominal pain upper	3.72	Product substitution issue	1.55	Therapy interrupted	3.27	Vomiting	5.65
Ketoacidosis	2.78	Fatigue	3.62	Acute kidney injury	1.52	Diarrhoea	3.22	Blood bilirubin increased	5.65
Pruritus	2.71	Dyspepsia	3.12	Blood pressure increased	1.49	Hospitalisation	3.19	Rash	5.65
Diarrhoea	2.39	Weight decreased	3.02	Rash	1.46	Blood alkaline phosphatase increased	2.84	Abdominal pain	4.84
Vomiting	2.13	Abdominal pain	2.77	Diarrhoea	1.45	Abdominal pain upper	2.83	Abdominal discomfort	4.03
Balanoposthitis	2.09	Injection site erythema	2.69	Hypertension	1.42	Malaise	2.61	Blood glucose increased	4.03
Fatigue	1.88	Injection site pruritus	2.65	Arthralgia	1.41	Headache	2.45	Flatulence	3.23
Glycosylated haemoglobin increased	1.68	Abdominal discomfort	2.51	Malaise	1.39	Insomnia	2.13	Blood alkaline phosphatase increased	3.23
Dehydration	1.66	Weight loss poor	2.51	Hyperkalaemia	1.34	Abdominal distension	2.07	Back pain	3.23
Malaise	1.63	Malaise	2.45	Palpitations	1.05	Vomiting	2.02	Headache	3.23
Death	1.59	Eructation	2.44	Angioedema	1.03	Abdominal pain	1.98	Syncope	3.23

Abbreviations: ADR, adverse drug reaction.

### Serious ADRs of five anti-fibrotic agents

**FIGURE 2:**
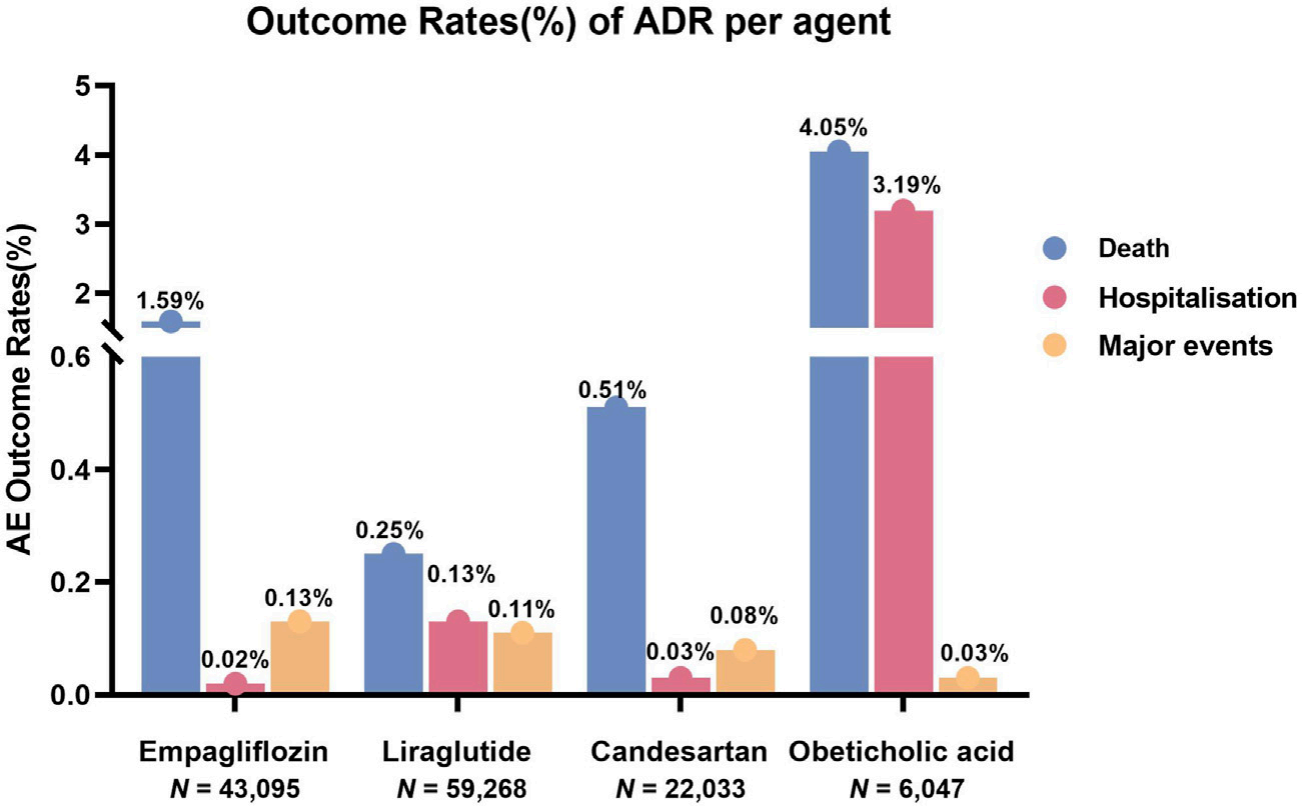
Outcomes for serious ADRs associated with five anti-fibrotic agents. Major events comprising life-threatening events, disability, and congenital anomaly.

Through the WHO-VigiAccess, we can also find major ADRs of anti-fibrotic agents, including life-threatening events, disability, and congenital malformations. The proportion of serious ADRs that occurred for empagliflozin, liraglutide, candesartan, obeticholic acid, and resmetirom was 1.74%, 0.48%, 7.28%, 0.62%, and 0%, respectively (Figure 2).

### Disproportionality analysis based on gastrointestinal disorders and related ADRs

**FIGURE 3:**
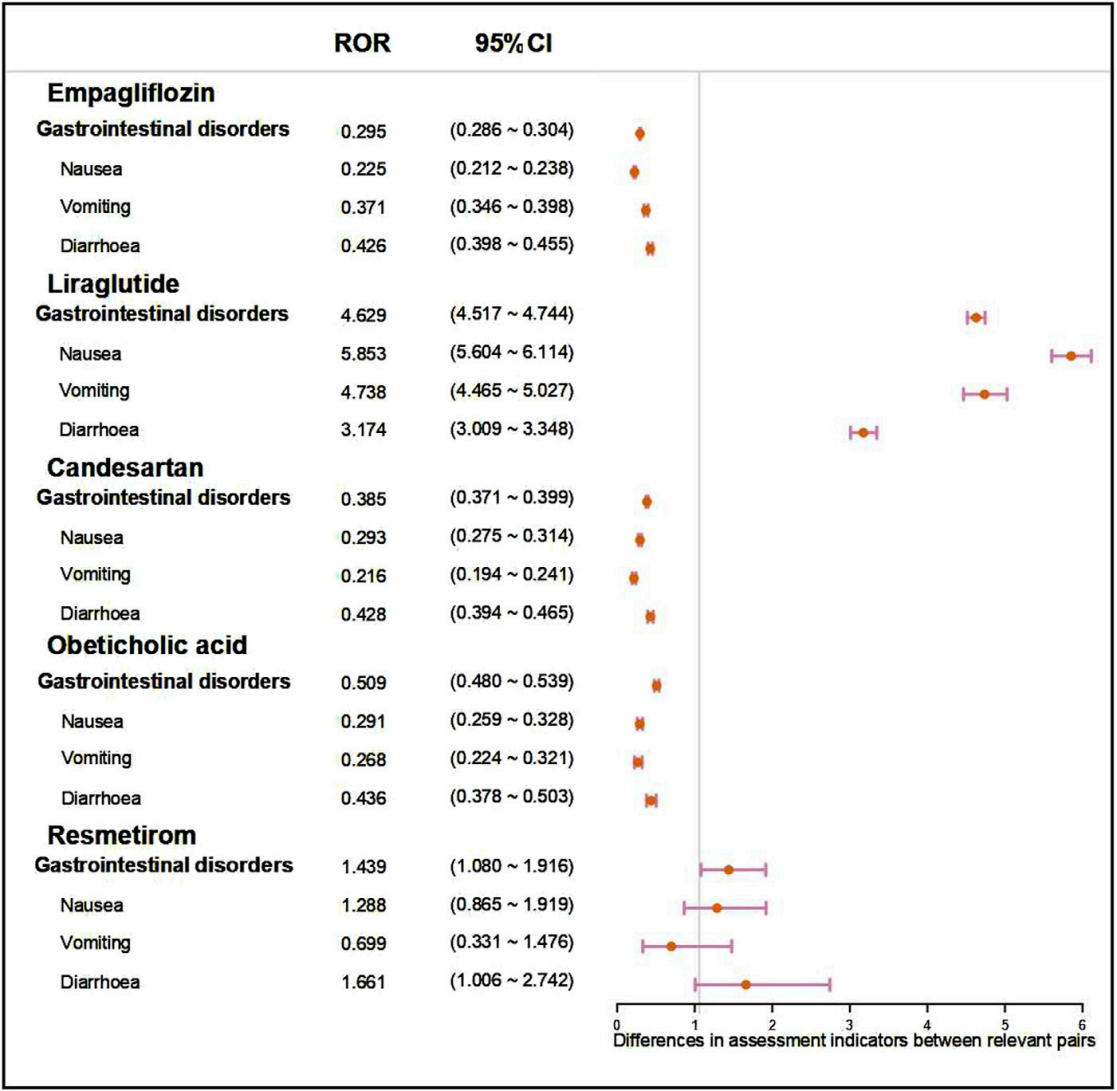
Forest plot showed the ROR value and 95%CI of five anti-fibrotic agents on gastrointestinal disorders and three most common symptoms (nausea, vomiting, diarrhoea). ROR, reporting odd ratio; 95% CI, 95% confidence interval.

By observing and comparing the SOC distributions of five anti-fibrotic agents, it was found that gastrointestinal disorder related symptoms were the most common ADR. To further compare these five agents, we performed a disproportionality analysis based on gastrointestinal disorders and three most common PTs (nausea, vomiting, diarrhoea). As shown in Figure 3, liraglutide might be associated with a greater risk of gastrointestinal disorder than the other agents with the ROR value of 4.629 (95% CI: 4.517–4.744). Moreover, liraglutide treatment also showed a significant risk of nausea (ROR: 5.853, 95% CI: 5.604–6.114), vomiting (ROR: 4.738, 95% CI: 4.465–5.027), and diarrhoea (ROR: 3.174, 95% CI: 3.009–3.348) compared to the other agents. Similar results were obtained assessed by the other metric, with the PRR value of liraglutide treatment was 3.566 (95% CI: 3.492–3.642) in gastrointestinal disorders, 5.392 (95% CI: 5.169–5.624) in nausea, 4.572 (95% CI: 4.313–4.847) in vomiting, 3.087 (95% CI: 2.929–3.252) in diarrhoea (Supplementary Figure S1).

## Discussion

The global health burden of liver fibrosis is increasing due to its progression to cirrhosis and HCC if untreated. While recent advancements have shed light on potential anti-fibrotic agents, the adverse effects pose significant challenges to their clinical applications. This study provides a comprehensive analysis of ADRs associated with hepatic anti-fibrotic agents currently under investigation in phase 3 and above clinical trials, using data derived from the WHO-VigiAccess database. By focusing on five promising agents, empagliflozin, liraglutide, candesartan, obeticholic acid, and resmetirom, the study demonstrated overall ADR profiles, the distribution of SOCs, and the disproportionality of gastrointestinal disorder related ADRs. These findings not only underscored the challenges in pharmacotherapy for liver fibrosis but also suggested critical considerations for clinical practice.

The five anti-fibrotic agents exhibit distinct safety profiles influenced by their pharmacological mechanisms, usage history, and targeted patients. SGLT2 inhibitors, including empagliflozin, are widely used as hypoglycemic drugs in T2DM patients, especially with renal impairment (Fioretto et al., 2016) for a considerable period. Liraglutide is an approved obesity pharmacotherapy with proven clinical effectiveness and long-term benefits (Davies et al., 2015; Papamargaritis et al., 2024). As a kind of ARB agent, candesartan is a long-established anti-hypertensive medication. These three agents demonstrated higher ADR report rates, attributed to their longer clinical use and broader patient base. In contrast, obeticholic acid and resmetirom, as novel anti-fibrotic agents, showed fewer reports yet revealed ADR patterns consistent with their pharmacological targets.

The study revealed a significant number of ADR reports across the five anti-fibrotic agents, with 130,567 reports. The predominance of ADRs is shown in females, possibly reflecting a greater propensity for women to report ADRs or sex-related pharmacokinetic differences. Age distribution indicated a peak in ADRs among individuals aged 45–64 years. Aging increases the likelihood of long-term exposure to liver-damaging conditions, and many liver diseases progress slowly, with fibrosis accumulating over decades, eventually leading to cirrhosis (Powell et al., 2021). Geographic variations in ADRs, with a majority from the Americas (53.38%) and Europe (31.05%), may reflect regional differences in drug availability, healthcare systems, and pharmacovigilance practices.

Specific agents exhibited distinct ADR profiles. Empagliflozin demonstrated unique risks, and several life-threatening events, including ketoacidosis, dehydration, and serious infections. SGLT2 inhibitors provide a protective effect on the kidneys via reduced transglomerular pressure and increased urinary glucose excretion in the renal proximal tubules (Saisho, 2020). However, glucosuria increases the risk of genitourinary tract infection and dehydration (Donnan et al., 2019), and glucagon secretion may lead to an overproduction of ketone bodies (Ogawa and Sakaguchi, 2016). Therefore, empagliflozin treatment requires close monitoring of blood glucose and ketone body levels in cirrhotic patients, particularly in those with comorbidities such as diabetes. Candesartan showed high rates of life-threatening events, most frequently associated with AKI. As a common anti-hypertensive drug, candesartan causes vasodilation of the renal efferent arteriole and results in the glomerular filtration rate reduction and ultimately AKI (Schoolwerth et al., 2001). The real-world data showed that the rising creatinine (>10%) after initiation of ARB treatment was associated with worse health outcomes (Fu et al., 2019). Therefore, candesartan should be used with caution for the treatment of liver fibrosis when patients suffer from renal insufficiency, and renal function (including serum creatinine and eGFR) monitoring is necessary during treatment. The relatively low serious ADR rates for obeticholic acid suggest a favorable risk profile in current clinical contexts. As the most advanced agent in FXR agonists, obeticholic acid showed beneficial effects on hepatic cholestatic fibrosis and has been approved as the second-line treatment for primary biliary cholangitis (Lindor et al., 2019). Resmetirom displayed minimal serious ADRs, and the most common adverse events were generally mild, transient diarrhea and nausea at treatment initiation (Harrison et al., 2019; Harrison et al., 2024). Therefore, it is promising to be the first FDA-approved treatment for liver fibrosis.

Gastrointestinal disorders were the most reported ADRs, accounting for nearly one-third of all cases (29.44%). This pattern aligns with the known mechanisms of hepatic anti-fibrotic agents, many of which target metabolic and inflammatory pathways closely linked to gastrointestinal physiology. The results of disproportionality analysis showed that liraglutide exhibited a significantly high risk of gastrointestinal ADRs, particularly nausea, vomiting, and diarrhoea. As a kind of GLP-1 analogue, it regulates gastric emptying and metabolic dysfunction that induced gastrointestinal symptoms. Clinical studies reported that gastrointestinal complaints often occurred within the first week of treatment, and most of the subjects reported ADRs of mild to moderate severity (Degn et al., 2004; Vilsbøll, 2007). Despite these risks, liraglutide remains a promising agent for liver fibrosis treatment. Thus, strategies to mitigate these side effects, such as dose titration and dietary modifications are required.

Currently, there are some agents in phase 2 or about to begin phase 3 clinical trials for the treatment of liver fibrosis with encouraging results. For example, aramchol is a partial inhibitor of the stearoyl-CoA desaturase 1 (SCD1) enzyme that regulates the fatty acid production process. In a phase 2 study, NASH resolution and liver fibrosis improvement were observed of aramchol (Ratziu et al., 2021; Ratziu et al., 2024). The direct anti-fibrotic drug pirfenidone which is approved for the treatment of idiopathic lung fibrosis (Lancaster et al., 2017) has been confirmed hepatoxic (Poo et al., 2020). However, hydronidone, a novel structural modification of pirfenidone, has showed less hepatoxicity. In a phase 2 study, hydronidone plus entecavir showed a significant reduction of liver fibrosis degree in patients with chronic hepatitis B (CHB). These are promising pharmacotherapies for liver fibrosis; however, as an investigational drug, the ADRs cannot be searched from the WHO-VigiAccess database at present.

The study analyzed the large dataset from a globally representative pharmacovigilance system, and it focused on agents in phase 3 and above clinical trials, which are pivotal in determining whether a new treatment is safe and effective for widespread use. The diverse safety profiles of these agents highlight the importance of personalized treatment strategies. Clinicians must weigh the therapeutic benefits against potential risks, particularly in populations at higher risk of serious ADRs (e.g., elders, patients with comorbidities). Regular biochemical and clinical evaluations can help detect early signs of complications, such as renal impairment and ketoacidosis. Moreover, gastrointestinal ADRs, while not life-threatening, can significantly impact patient adherence. Strategies to alleviate these side effects and concurrent use of supportive therapies should be explored in clinical practice.

This study has several limitations. First, as an SRS, the WHO-VigiAccess is inherently limited by reporting biases and underreporting. Moreover, the data relies on voluntary reporting may not fully capture the true scope of ADRs for anti-fibrotic agents. This is an inherent limitation in the context of clinical trials, especially when evaluating emerging therapies for liver fibrosis. When approved therapies become available, more comprehensive data on ADRs will be obtained through a mandatory reporting system. Second, the demographic skew towards the Americas and Europe in ADRs underscores the need for greater representation from other regions, particularly Asia and Africa, where liver fibrosis prevalence is high. Third, the long-term safety of hepatic anti-fibrotic agents remains uncertain. Longitudinal studies with diverse populations are essential to evaluate cumulative risks and rare ADRs.

The pipeline of hepatic anti-fibrotic agents offers immense promise, but achieving a balance between efficacy and safety remains challenging. Incorporating real-world evidence, as demonstrated in this study, into drug development processes can help identify safety signals early, and guide the design of phase 4 trials and future markets. Moreover, combination therapies targeting multiple pathways in liver fibrogenesis may enhance while reducing dose-dependent risks of ADRs. For instance, pairing agents with complementary mechanisms could achieve synergistic effects with fewer ADRs.

## Conclusion

The safety profiles of hepatic anti-fibrotic agents present both opportunities and challenges for clinical practice and drug development. While these agents showed promise in treating liver fibrosis, their ADR profiles need careful consideration in clinical practice. Future efforts should focus on improving the pharmacovigilance system, expanding population diversity in trials, and conducting ongoing research and rigorous post-marketing surveillance. By addressing these challenges, we can achieve safer and more effective therapies for liver fibrosis, ultimately improving outcomes for patients worldwide.

## Data Availability

The original contributions presented in the study are included in the article/Supplementary Material, further inquiries can be directed to the corresponding author.
