# Supplementary figures and images for: Adverse events of hepatic anti-fibrotic agents in phase 3 and above clinical trials: a descriptive analysis of the WHO-VigiAccess database
Source: Front Pharmacol. 2025 Jan 24;16:1534628. doi: 10.3389/fphar.2025.1534628 (PMC11802529; doi:10.3389/fphar.2025.1534628)

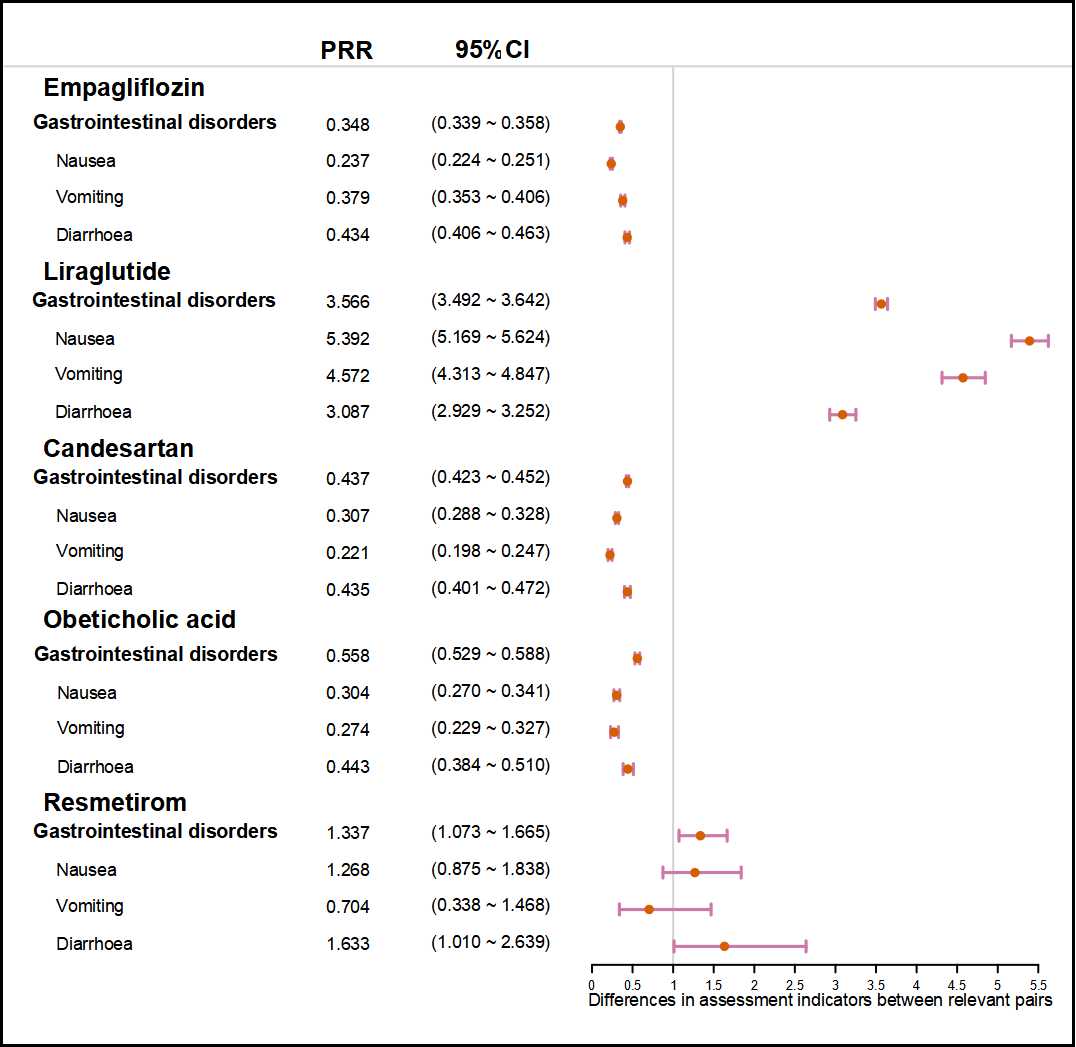

Supplement: Supplementary file 1 [file Image1.jpeg]
